# Restraint care in the pediatric intensive care unit: a qualitative investigation of nurses’ perspectives and experiences

**DOI:** 10.3389/fped.2026.1825957

**Published:** 2026-05-29

**Authors:** Xiaowen Dai, Qingqing Liu, Shuyi Ma, Li Wei

**Affiliations:** PICU, Children’s Hospital of Nanjing Medical University, Nanjing, Jiangsu Province, China

**Keywords:** care, nurse, pediatric intensive care unit, physical restraint, qualitative research

## Abstract

**Background:**

Restraint use in pediatric intensive care units (PICUs), while essential for preventing unplanned extubation, presents ethical and practical challenges. Nurses, as primary implementers, often face psychological tensions arising from their dual role as caregivers and enforcers of safety. Yet, their subjective experiences remain underexplored.

**Methods:**

A descriptive phenomenological design was employed. Purposive sampling recruited 16 PICU nurses from a tertiary children's hospital in Jiangsu, China, between March and June 2025. Semi-structured, face-to-face interviews (30–60 min) were conducted and analyzed using Colaizzi's method. Ethical approval was obtained, and triangulation ensured rigor. Sample size was determined by data saturation.

**Results:**

Sixteen PICU nurses participated. Five core themes emerged: (1) ethical tensions in balancing patient safety with respect for autonomy; (2) practical difficulties due to ill-fitting restraint tools and technical demands; (3) nurse-family communication breakdowns from emotional resistance and information asymmetry; (4) cumulative psychological strain, including vicarious trauma and diminished professional identity; (5) institutional needs for clear protocols, targeted training, and staffing support. Practice gaps included inconsistent emergency assessments, incomplete family consent, poor site-monitoring compliance, and lack of pediatric-specific tools.

**Conclusion:**

PICU nurses face multifaceted challenges in restraint care. Improvements require standardized pediatric guidelines, communication-focused training, non-restraint alternatives, and enhanced institutional support.

## Introduction

The Pediatric Intensive Care Unit (PICU) is a specialized setting for the concentrated treatment of critically ill children, where nursing practice revolves around two core objectives: “life support” and “safety assurance” (1). Due to their young age, limited cognitive abilities, and severe medical conditions, children in the PICU frequently require invasive procedures such as endotracheal intubation and central venous catheterization, rendering them significantly more vulnerable to adverse events—including unplanned extubation, falls, and self-injury—compared to adult patients (2). The incidence of unplanned extubation among these patients ranges from 8.3% to 15.6% (3, 4). Notably, such events are associated with prolonged mechanical ventilation, an average increase of 3.2 days in hospital stay, and a 2.1-fold higher mortality rate (5). Physical restraint is defined as the use of manual or mechanical devices to limit a child's voluntary physical movement to prevent harm to themselves or interference with medical treatments (6). Restraint care is widely employed as a key measure to prevent adverse events; it involves limiting children's physical movement through physical or pharmaceutical means to ensure the smooth progression of treatment.

However, physical restraint in pediatric intensive care is inherently intertwined with profound ethical controversies and practical contradictions. The World Health Organization (WHO) explicitly stipulates in its Patient Safety Goals that restraint measures should be regarded as a “last resort,” emphasizing the imperative to balance the ethical principles of “minimizing harm” and “maximizing benefit” (7). Clinically, inappropriate restraint application may lead to a range of adverse outcomes in children, including skin injuries, limb dysfunction, and psychological trauma such as anxiety and fear (8). As direct implementers of restraint care, PICU nurses occupy a dual role: they serve as both guardians of medical safety and bearers of ethical dilemmas (9). This duality compels them to mitigate clinical risks through restraint while concurrently confronting children's distressed responses and families’ skepticism or resistance (10, 11). The psychological conflict and practical confusion arising from this dual role directly hinder the standardized implementation and quality improvement of restraint care in PICU settings (12). Notably, distinct contextual factors in Chinese PICUs—including higher nurse-to-patient ratios (1:4–5 vs. 1:2–3 in many Western settings), limited availability of child life specialists and distraction tools, strong family expectations for direct care participation, and the absence of standardized ethical decision-making training—further complicate restraint practice and underscore the need for context-specific research. At the policy level, China's National Health Commission (NHC) issued general patient safety guidelines in 2021 that classify restraint as a “last resort” but lack PICU-specific operational details, leading to practical implementation challenges.

Existing studies on pediatric restraint care have predominantly focused on evaluating intervention effectiveness (e.g., incidence of adverse events) or investigating family attitudes, with relatively limited exploration of nurses’ subjective experiences (13, 14). While international qualitative research has identified “moral distress” among pediatric nurses engaged in restraint practices (15–17), these findings cannot be directly generalized to the Chinese context due to fundamental differences in cultural backgrounds, healthcare systems, and clinical environments. Moreover, the three qualitative studies available in China concerning pediatric restraint have centered on family attitudes and intervention strategies, rather than on nurses’ lived experiences (18–20). Given this gap, the present study aimed to explore the authentic, subjective experiences of PICU nurses. A descriptive phenomenological design was adopted to address the following objectives: (1) revealing nurses’ practical dilemmas and psychological perceptions in restraint care; (2) analyzing key factors influencing the quality of restraint care in Chinese PICU settings; and (3) providing empirical evidence to inform the formulation of contextually appropriate PICU restraint care guidelines in China.

## Materials and methods

### Study design

This study employed a descriptive phenomenological design to explore the essential structure and lived experiences of nurses engaging in restraint care within the PICU. The theoretical framework was Colaizzi's descriptive phenomenological approach (Colaizzi, 1978), which is specifically tailored to uncover the inherent meaning of human experiences through systematic inquiry. Colaizzi's methodology was selected for its emphasis on grounding findings in participants’ subjective narratives rather than testing pre-conceived hypotheses.

### Ethical approval

This study was approved by the Ethics Committee of the Children's Hospital of Nanjing Medical University (Ethics No.: 202509006-1) and strictly adhered to the principles of the Declaration of Helsinki. All participants provided written informed consent, which explicitly included consent for audio recording of interviews. To safeguard the anonymity and confidentiality of participants, the following strategies were implemented: (1) All personal identifiers (names, employee numbers, patient information) were completely removed from transcribed data; (2) Participants were assigned unique codes (N1–N16) for all study documents to replace any personal identifiers; (3) Audio recordings and transcribed texts were stored in a password-protected computer with restricted access limited exclusively to members of the research team; (4) Hard copies of consent forms and research records were stored in a locked filing cabinet in the research team's office, with access restricted to the principal investigator. Additionally, all members of the research team signed a confidentiality agreement to ensure strict compliance with ethical requirements regarding participant data protection. Audio recordings will be retained for 5 years after publication and then permanently deleted.

### Participants and setting

The study population consisted of registered nurses working in the PICU of a tertiary A children's hospital in Jiangsu Province, China, who had experience in implementing restraint care. Purposive sampling with maximum variation was used, stratifying by age, years of PICU experience, educational background, professional title, and marital status to capture diverse perspectives. This hospital serves as a regional critical care center, admitting over 1,500 critically ill children annually.

Inclusion criteria were: (1) Registered nurses with valid practicing certificates; (2) continuous PICU work experience of ≥1 year and independent performance of at least 20 restraint care cases; (3) voluntary participation with signed informed consent; (4) ability to express themselves clearly. Exclusion criteria were: (1) trainee or rotating nurses; (2) nurses who had experienced restraint-related adverse events in the preceding 3 months (to minimize the potential impact of acute emotional trauma on narratives of long-term lived experiences); (3) nurses with mental illness or communication disorders.

Sample size was determined by data saturation, defined as the point at which three consecutive interviews yielded no new themes or sub-themes. Saturation monitoring and confirmation procedures are described in the Results section.

### Data collection

#### General information questionnaire

A self-designed questionnaire collected demographic information (age, years of nursing experience, educational background, professional title, marital status, and whether they have children) and occupation-related information (frequency of performing restraint care per month, whether they had received specialized restraint care training).

#### Semi-structured interview guide

The interview guide was developed through a four-stage process: (1) a literature review of 32 relevant articles (2019–2024) retrieved from PubMed, CNKI, and CINAHL; (2) drafting of 8 core questions covering decision-making, implementation challenges, family communication, psychological impacts, and improvement needs; (3) expert consultation with three PICU nursing experts (≥10 years’ experience) and two qualitative research experts (≥5 years’ phenomenological experience), who refined the questions for clarity and depth; (4) pre-interviews with two eligible PICU nurses (who were not included in the main study) to adjust question order and probes. The final guide focused on six domains: lived experiences and associated emotions; factors influencing restraint decision-making; practical challenges during implementation; nurse-family communication; psychological impacts of long-term restraint care; and suggestions for improvement. All questions adopted an open-ended, phenomenological approach.

#### Interview procedure

All interviews were conducted face-to-face in a quiet PICU meeting room, lasted 30–45 min, and were audio-recorded with permission. Non-verbal information was documented in real time. To maintain reflexivity, interviewers documented personal biases in advance, emphasized their researcher (not supervisory) role, and completed reflexive journals after each session. Within 24 h, two researchers independently transcribed the audio recordings and conducted back-to-back verification to ensure ≥98% transcription accuracy.

#### Data analysis and rigor

Data analysis strictly followed Colaizzi's seven-step method, conducted manually to enhance deep engagement with the data. Bracketing (epoché) was systematically implemented: prior to data collection, all researchers individually documented their preconceptions about PICU restraint care, which were then shared and discussed to foster collective awareness. These bracketing notes were regularly cross-referenced during analysis to ensure interpretations remained grounded in participants’ narratives.

All interviews were conducted in Chinese. Transcription, coding, and initial theme development were performed in the original language to preserve semantic accuracy. For publication, themes and representative quotes were translated into English by a bilingual research team member, followed by independent back-translation; discrepancies were resolved through discussion.

The specific analytical steps were: (1) all researchers repeatedly read the transcripts and field notes; (2) meaningful statements related to the research theme were extracted; (3) each meaningful statement was independently coded by two researchers, with coding memos documenting the rationale; (4) codes with similar meanings were grouped into sub-themes; (5) sub-themes were synthesized into core themes based on their shared essence; (6) a detailed description of each core theme was prepared; (7) member checking was conducted with all participants. Disagreements in coding (12% of initial codes) were resolved through team discussion. Triangulation (method, researcher, and theoretical) was employed to enhance credibility, alongside an audit trail, detailed contextual descriptions, and regular team reviews, all under the guidance of a qualitative research expert. The completed SRQR checklist is provided as Supplementary File 2.

## Results

### Characteristics of participants

A total of 16 PICU nurses were included. Data saturation was initially reached after the 10th interview, with no new sub-themes emerging from the 11th and no new meanings from the 12th. Three additional confirmatory interviews (N13–N15) yielded no new insights, and a final 16th interview further validated saturation. Table 1 displays participants’ demographic characteristics. Ages ranged from 23 to 42 years, and length of nursing experience spanned 2 to 18 years (mean 7.8 ± 3.7 years). Twelve held a bachelor's degree and four an associate degree. Regarding professional titles, 3 were charge nurses, 8 were senior nurses, and 5 were junior nurses. Ten participants were married, and six of these had children.

**Table 1:** Demographic characteristics of participating PICU nurses (*n* = 16).

Characteristic	Distribution
Age (years)	Range: 23–42; Mean ± standard deviation: 31.5 ± 5.3
Length of PICU nursing experience (years)	Range: 2–18; Mean ± standard deviation: 7.8 ± 3.7
Educational background	Bachelor's degree: 12 nurses (75.0%) Associate degree: 4 nurses (25.0%)
Professional title	Charge nurse: 3 nurses (18.8%) Senior nurse: 8 nurses (50.0%) Junior nurse: 5 nurses (31.2%)
Marital status	Married: 10 nurses (62.5%) Unmarried: 6 nurses (37.5%)
Married with children	6 nurses (among married participants)

### Current Status of restraint care practices

Table 2 summarizes the restraint care practices of the 16 participants. A large majority reported performing pre-restraint indication assessment; however, four noted skipping assessments during emergencies. A large majority obtained family informed consent, although two failed to do so due to family emotional agitation. A large majority selected appropriate restraint tools, but six identified a lack of specialized straps for infants. Fewer than half reported monitoring restrained sites every 15 min, citing difficulty when caring for multiple children. More than half documented restraint care, though records focused primarily on restraint status rather than dynamic changes in comfort. A minority implemented humanistic care measures during restraint, with only three reporting soothing children throughout the process. More than half considered non-restraint alternatives, most commonly family companionship and toy distraction.

**Table 2:** Restraint care practices among the 16 participating nurses.

Practice item	Number of nurses with standard implementation	Descriptive frequency term	Key issues described
Pre-restraint indication assessment	13	A large majority of participants	4 nurses reported “skipping assessment during emergency situations”
Obtaining informed consent from families	14	A large majority of participants	2 nurses failed to obtain consent due to “family emotional agitation”
Selection of appropriate restraint tools	12	A large majority of participants	6 nurses noted “a lack of specialized restraint straps for infants”
Monitoring of restrained sites every 15 min	9	Fewer participants	“Difficulty maintaining frequency when caring for multiple children” (N6)
Documentation of restraint care processes	11	More than half of participants	Documentation primarily focused on “restraint status” with insufficient dynamic assessment
Implementation of humanistic care measures	7	A minority of participants	Only 3 nurses reported “soothing children by patting during restraint”
Consideration of alternative measures to restraint	10	More than half of participants	Common alternatives included “family companionship” and “distraction with toys”

### Core themes

**Figure 1:**
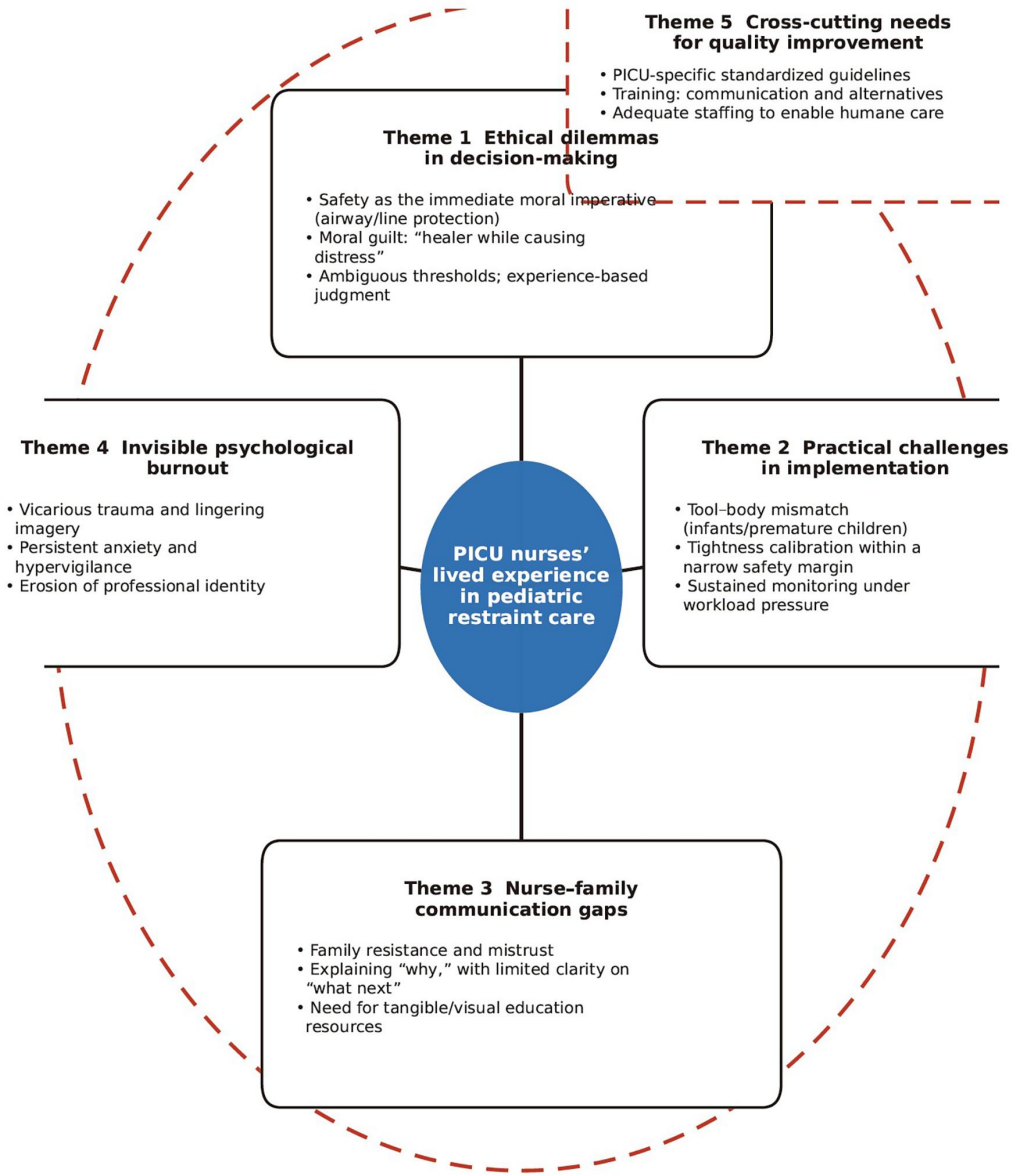
Thematic Map illustrating the hierarchical and interactive relationships Among core themes and Sub-themes of PICU nurses’ lived experiences in pediatric restraint care.

Through Colaizzi’s phenomenological analysis, five core themes and 12 sub-themes were extracted, as depicted in the thematic map (Figure 1).

#### Theme 1: ethical dilemmas in restraint decision-making—the existential tension of being a “healer who causes distress”

This theme captures the core ethical paradox nurses face between protecting children from treatment-related harm and upholding their identity as compassionate caregivers. A large majority of participants (*N* = 14) identified pipeline safety as the primary consideration for restraint (e.g., “When I see a child grabbing the endotracheal tube, my first reaction is that restraint is necessary”) (Table 3). However, the absence of objective criteria for assessing agitation or cognitive status left nurses relying on subjective judgment. Many participants (*N* = 11) reported profound moral guilt (e.g., “I’m clearly trying to save him, but it feels like I’m hurting him”), a feeling particularly pronounced among married nurses with children (5/6). Half of the participants (*N* = 8) indicated that they based restraint decisions solely on personal experience, highlighting the lack of clear institutional guidelines.

**Table 3:** Factors influencing ethical dilemmas in restraint decision-making with illustrative quotes.

Influencing factor	Descriptive frequency term	Typical statement
Pipeline safety risk	A large majority of participants (*N* = 14)	“Extubation could be fatal; restraint is imperative” (N3)
Child's distress response	A large majority of participants (*N* = 12)	“The child cried hysterically—I almost released the restraint” (N2)
Family attitude	More than half of participants (*N* = 10)	“We hesitate to restrain if families disagree” (N9)
Lack of clear standards	Half of the participants (*N* = 8)	“Textbooks say ‘restrain when necessary,’ but how is ‘necessary’ defined?” (N5)
Insufficient ethical training	A minority of participants (*N* = 5)	“We were never taught how to balance safety and ethics in school” (N1)

#### Theme 2: practical challenges in restraint implementation—conflicts between tool limitations and technical precision requirements

This theme describes operational barriers rooted in the mismatch between available resources and children's specialized needs. Many participants (*N* = 11) noted that existing restraint tools were designed for adult anatomy and ill-suited for infants (e.g., “The smallest restraint belt is still too loose for premature infants”), with four reporting skin redness from ill-fitting devices. A large majority (*N* = 13) identified adjusting strap tightness as a critical technical challenge (e.g., “Loosening by 1 cm risks extubation; tightening by 1 cm risks pressure injury”), requiring repeated adjustments. Compounding these issues, a large majority (*N* = 12) described being chronically overworked, with heavy patient loads preventing optimal monitoring (e.g., “Caring for 6 children alone, just checking restraints takes half an hour”).

#### Theme 3: cognitive gaps in nurse-family communication—barriers rooted in information asymmetry and emotional opposition

This theme explores communication challenges stemming from a lack of shared understanding and unaddressed emotional needs. A large majority of participants (*N* = 13) reported encountering family resistance to restraint (e.g., “Families accused us of ‘being lazy and not watching the child’”), which was more prevalent among parents under 30. More than half (*N* = 10) stated they could only explain the rationale for restraint but could not provide details such as expected duration or comfort-alleviating strategies, owing to the absence of standardized communication scripts. To address this, a large majority (*N* = 12) called for visual educational resources such as graphic manuals (e.g., “Words alone don't work; families need to see ‘other children go through this too’”).

#### Theme 4: invisible burnout in professional psychology—erosion of professional identity amid chronic emotional burden

This theme captures the psychological toll of long-term restraint care. A large majority (*N* = 14) reported experiencing empathic distress and vicarious trauma (e.g., “Watching children struggle gives me nightmares”), which was especially prominent among nurses with less than 5 years of PICU experience (5/5). A large majority (*N* = 13) reported persistent anxiety (e.g., “I’m always afraid something will go wrong with restraints”). Over time, more than half (*N* = 10) felt that their professional identity had been diminished (e.g., “more like guards than nurses”), with 3 nurses having considered leaving the PICU.

#### Theme 5: diverse demands for quality improvement—expectations for institutional support and resource optimization

This cross-cutting theme reflects nurses’ calls for systemic change. All participants (*N* = 16) desired standardized, PICU-specific restraint guidelines (e.g., “Step-by-step instructions from assessment to removal”). A large majority (*N* = 13) requested specialized training in family communication and non-restraint alternatives, and a large majority (*N* = 12) highlighted the need for increased human resources to permit closer monitoring and humanistic care.

## Discussion

This study reveals that PICU nurses face profound ethical dilemmas, practical barriers, communication gaps, and psychological strain in restraint care, all of which demand systemic improvements.

### Ethical dilemmas and moral distress

The prioritization of pipeline safety reported by the majority of participants (*N* = 14) reflects a “safety-first” professional pressure prevalent in China's medicolegal environment, which can drive risk-avoidant decision-making and marginalize children's comfort. This finding resonates with Jameton's concept of moral distress—knowing the ethically appropriate action but being constrained from taking it (21, 22). The moral guilt expressed by many nurses (*N* = 11) mirrors the conflict between beneficence and respect for autonomy (23, 24). To address this ethical ambiguity, we recommend adopting a standardized “restraint decision tree” (e.g., as proposed by the Pediatric Nursing Association), which translates vague judgments into a structured, three-tier assessment of risk level, alternative measures, and restraint planning. Integrating case-based training into regular staff meetings would facilitate adoption without excessive workload (25–28).

### Practical challenges and tool misalignment

The poor fit of adult-oriented restraint tools for children underscores a lag in pediatric-specific device innovation. Consistent with prior evidence that less than 50% of tools are adaptable to pediatric patients (29), ill-fitting devices compromise both safety and comfort. From Watson's caring theory, such tool-patient mismatch undermines the “caring moment” (30, 31). We propose the development of age-specific restraint systems through nursing-engineering collaboration, such as silicone “nest-style pads” for premature infants and “smart straps” with pressure sensors. A phased implementation—starting with low-cost modifications to existing straps—could mitigate financial barriers, and a standard operating procedure with quantifiable tightness criteria would reduce technical uncertainty (32).

### Nurse-Family communication gaps

Family resistance, identified by most participants (*N* = 13), illuminates the breakdown of relational ethics in pediatric care. When nurses’ explanations focus only on the rationale for restraint without addressing the child's comfort or the family's emotional distress, communication becomes transactional rather than relational. The call for visual educational resources (*N* = 12) indicates that families require concrete, empathetic information to build trust. We suggest developing standardized communication scripts that include comprehensive restraint information and integrating brief (15-minute), biweekly scenario-based empathy training into existing staff meetings, supplemented by pre-printed visual manuals to reduce nurses’ preparation time (33, 34).

### Psychological strain and moral residue

The vicarious trauma, pervasive anxiety, and diminished professional identity reported by participants reflect an “invisible burnout” rooted in moral residue—the accumulated emotional toll of unresolved moral distress (35). The feeling of being “more like guards than nurses” signals a crisis of professional meaning. Addressing this requires both individual and systemic support: brief “moral check-ins” during shift handovers could provide immediate debriefing, while clinical nurse specialists could offer targeted support for nurses experiencing significant distress. Reducing the systemic drivers of moral distress—through clearer guidelines and adequate staffing—remains a priority.

### Systemic demands and cross-disciplinary insights

Nurses’ unanimous call for standardized guidelines (*N* = 16), training (*N* = 13), and increased staffing (*N* = 12) aligns with Leininger’s Culture Care Theory, which emphasizes adapting care to the local cultural and systemic context (36–38).

In Chinese PICUs, with nurse-to-patient ratios of 1:4–5, understaffing directly impedes close monitoring and humanistic care. Short-term solutions such as flexible float pools could alleviate peak workload pressures. Interestingly, pediatric dentistry has developed evidence-based behavior management guidelines that may offer transferable communication and desensitization strategies; however, the fundamental differences between elective dental procedures and life-sustaining critical care limit direct application. This cross-disciplinary parallel merits further exploration and could inform future nursing curricula.

## Feasibility and limitations

The proposed interventions must address cost, protocol resistance, and training time constraints. Engaging frontline nurses in guideline development can foster ownership and pragmatic adaptation. Institutional commitment to dedicated funding and protected training time will be crucial facilitators. Several limitations should be considered: the single-center design may limit transferability; the exclusive focus on nurses omits physician, child life specialist, and family perspectives; and organizational factors were not systematically explored. Future research should adopt multi-center, multi-stakeholder designs and explore longitudinal trajectories of moral distress.

## Conclusion

This study illuminates the multifaceted challenges PICU nurses face in restraint care, revealing an interconnected web of ethical dilemmas, practical hurdles, communication gaps, and psychological strain. The pervasiveness of safety-prioritized decision-making, compounded by inadequate pediatric-specific tools, underdeveloped communication frameworks, and unsustainable staffing, highlights a critical gap between current practice and the ideal of balanced, humanistic care. Nurses’ calls for standardized guidelines, specialized training, and enhanced staffing reflect systemic rather than individual shortcomings. Addressing these requires a holistic approach encompassing age-specific restraint tool development, structured ethical decision-making frameworks, and communication training that addresses both informational and emotional needs. Such measures are essential to mitigate nurses’ moral distress, improve care quality, and uphold the rights and well-being of critically ill children.

## Data Availability

The raw data supporting the conclusions of this article will be made available by the authors, without undue reservation.
