# Supplementary material for: Restraint care in the pediatric intensive care unit: a qualitative investigation of nurses’ perspectives and experiences
Source: Front Pediatr. 2026 May 29;14:1825957. doi: 10.3389/fped.2026.1825957 (PMC13260538; doi:10.3389/fped.2026.1825957)
Supplement: Supplementary file 1 [file Supplementaryfile1.docx]

**Supplementary File 1: General Information Questionnaire and Semi-structured Interview Guide**

1. General Information Questionnaire

- Age

- Gender

- Total nursing work experience (years)

- PICU nursing work experience (years)

- Educational background

- Professional title

- Marital status

- Whether having children

- Frequency of performing restraint care per month

- Whether having received specialized training on restraint care

**2. Semi-structured Interview Guide**

Development process: This interview guide was developed through four stages, including literature review (32 relevant articles retrieved from PubMed, CNKI, and CINAHL, 2019–2024), initial draft formulation, expert consultation (3 PICU nursing experts with ≥10 years of experience and 2 qualitative research experts with ≥5 years of phenomenological research experience), and pre-interviews (2 eligible PICU nurses). Revisions such as merging overlapping questions, refining vague terms, and optimizing question order and follow-up probes were conducted to ensure the guide’s rigor, relevance, and clarity.

Core questions and follow-up probes:

1. Can you describe a memorable experience of providing restraint care for a pediatric patient? What emotions did you feel at that time?

Follow-up probes: What specific details of this experience left the deepest impression on you? How did you respond during this process?

2. What specific factors do you consider when deciding to implement restraint on a pediatric patient? How do these factors influence your decision-making?

Follow-up probes: Are there any conflicting factors in the decision-making process? How do you handle such conflicts?

3. What specific challenges have you encountered during the implementation of restraint care? What impacts do these challenges have on the quality of nursing care?

Follow-up probes: How have you tried to address these challenges? Were these attempts effective?

4. What situations usually occur when communicating with the family members of pediatric patients about restraint care? How do you respond to these situations?

Follow-up probes: Have you encountered family opposition to restraint? How did you handle it and what emotions did you feel at that time?

5. What impacts has long-term engagement in restraint care work had on your psychological state or professional cognition?

Follow-up probes: What did this experience mean for your understanding of your role as a PICU nurse? Have you adjusted your work attitude or behavior due to these impacts?

6. In your opinion, what aspects most need improvement in current restraint care practice? What kind of support do you hope to obtain?

Follow-up probes: Do you have any specific suggestions for optimizing restraint care processes or training? Who do you think should provide the support you need?
